# Epstein-Barr virus is present in the brain of most cases of multiple sclerosis and may engage more than just B cells

**DOI:** 10.1371/journal.pone.0192109

**Published:** 2018-02-02

**Authors:** Asma Hassani, John R. Corboy, Suhail Al-Salam, Gulfaraz Khan

**Affiliations:** 1 Department of Microbiology and Immunology, College of Medicine and Health Sciences, Tawam Hospital Campus, United Arab Emirates University, Al Ain, UAE; 2 Department of Neurology, University of Colorado School of Medicine, Rocky Mountain MS Center at University of Colorado, Aurora, United States of America; 3 Department of Pathology, College of Medicine and Health Sciences, Tawam Hospital Campus, United Arab Emirates University, Al Ain, UAE; University of Nebraska-Lincoln, UNITED STATES

## Abstract

Multiple sclerosis (MS) is a chronic neuroinflammatory condition of the central nervous system (CNS). It is a major cause of neurological disability in young adults, particularly women. What triggers the destruction of myelin sheaths covering nerve fibres is unknown. Both genetic and infectious agents have been implicated. Of the infectious agents, Epstein-Barr virus (EBV), a common herpesvirus, has the strongest epidemiological and serological evidence. However, the presence of EBV in the CNS and demonstration of the underlying mechanism(s) linking EBV to the pathogenesis of MS remain to be elucidated. We aimed at understanding the contribution of EBV infection in the pathology of MS. We examined 1055 specimens (440 DNA samples and 615 brain tissues) from 101 MS and 21 non-MS cases for the presence of EBV using PCR and EBER-*in situ* hybridization (EBER-ISH). EBV was detected by PCR and/or EBER-ISH in 91/101 (90%) of MS cases compared to only 5/21 (24%) of non-MS cases with other neuropathologies. None of the samples were PCR positive for other common herpesviruses (HSV-1, CMV, HHV-6). By quantitative PCR, EBV viral load in MS brain was mainly low to moderate in most cases. However, in 18/101 (18%) of MS cases, widespread but scattered presence of EBV infected cells was noted in the affected tissues by EBER-ISH. Immunohistochemical analysis of EBV gene expression in the 18 heavily infected cases, revealed that the EBV latent protein EBNA1, and to a lesser extent the early lytic protein BZLF1 were expressed. Furthermore, using double-staining we show for the first time that astrocytes and microglia, in addition to B-cells can also be infected. To the best of our knowledge, this is the most comprehensive study demonstrating that EBV is present and transcriptionally active in the brain of most cases of MS and supports a role for the virus in MS pathogenesis. Further studies are required to address the mechanism of EBV involvement in MS pathology.

## Introduction

Over 2 million people worldwide suffer from multiple sclerosis (MS), a debilitating neurological disease of autoimmune nature [1]. In MS, multiple lesions are formed in the brain and spinal cord as a result of continuous destruction of myelin sheaths surrounding the nerve fibers [2]. What initiates myelin destruction in the central nervous system (CNS) remains unknown [3]. It is believed that the immune system is involved in the development of MS in genetically predisposed persons who are exposed to certain environmental stimuli [4,5]. A growing body of evidence points to one common environmental stimulus, Epstein-Barr virus (EBV) infection [6–9].

EBV is a member of the herpes family of viruses that are common in humans. The silent infection and the life-long persistence are the keys to the widespread infection of EBV in the human population [10]. In most individuals, EBV infection occurs early in childhood and produces no symptoms. By contrast, delayed primary infection during adolescence can lead to infectious mononucleosis (IM) [11]. EBV primarily targets and remains latent in memory B cells [12,13]. Depending on the type of latency, EBV expresses different sets of latent products. In type III latency, typically seen in IM, up to 12 viral products, including 6 EBV-encoded nuclear antigens (EBNA-LP, EBNA1, EBNA2, EBNA3A, EBNA3B, and EBNA3C), 3 latent membrane proteins (LMP1, LMP2A, and LMP2B) and large quantities of 2 non-coding RNAs (EBERs) are expressed [14]. A small percentage of latently infected cells intermittently enter the lytic cycle resulting in the release of viral particles [15]. This transition from latency to lytic cycle is triggered by the expression of two immediate early viral proteins, BZLF1 and BRLF1 [16–18]. However, in newly infected B-cells, although BZLF1 is expressed, EBV lytic cycle is not induced [17]. It seems that additional conditions, such as, unmethylation of EBV genome and methylation of promoters of certain genes transactivated by BZLF1 are required for a complete successful lytic cycle and production of new virions [17,19]. For this reason, the expression of BZLF1 does not necessarily translate into viral shedding.

EBV is well-known for its intimate relationship with the immune system [20]. It is generally held that some EBV-associated pathologies result from the disruption of the virus-host immune system balance, and clinical manifestations of EBV infection emerge as a result of provoked immune response rather than of EBV itself [21]. In this context, several groups have shown that in MS, the immune response directed towards EBV is disrupted [22–28]. An expansion in humoral response to EBNA1 has been implicated in increasing the risk of developing MS [29,30]. In line with this observation, individuals who have increased antibody response to EBNA1 have higher odds ratio of developing MS compared to those with baseline IgG titers to EBNA1 [31]. Moreover, epidemiological studies have shown that almost all MS patients are infected with EBV compared to ~95% non-MS controls [32,33]. Thus, EBV seronegative individuals have almost zero risk of developing MS. However, a dramatic upsurge in MS risk is seen when these individuals seroconvert following EBV infection [34]. Furthermore, individuals who have a history of EBV-associated IM have also been shown to be at increased risk of developing MS [35,36].

In order to show any etiological link between EBV and the pathogenesis of MS, demonstrating the presence of the virus in the involved tissues is essential. Although several studies have investigated this, some even involving reasonably large number of cases [37], inter-laboratory variations in terms of technical and practical approaches, and region of the brain being examined, probably contribute to the conflicting results [6,38]. Whilst one group reported non-selective presence of EBV in MS and control brain tissues using PCR [39,40], two others could not detect viral RNAs in the brain using *in situ* hybridization [41,42]. More recent studies attempted combining different molecular and histochemical techniques to resolve the previous unclear results. Again some have reported negative findings [37,43,44], whilst others demonstrated the presence of EBV in MS demyelinated lesions and correlated viral infection with MS histopathological phenotypic traits [26,45–47]. Owing to the great heterogeneity of the brain, the molecular and cellular environment of one region does not necessarily represent another adjacent region, even in the same tissue block [6,38]. Thus, the absence of EBV in one region of the brain, cannot be interpreted to mean that the virus is absent from all parts of the brain. We hypothesized that EBV is widely present in MS brains, but a large sample size and thorough examination using a combination of sensitive techniques are required to demonstrate it.

Using PCR, we have previously shown that EBV is present in the meninges of about 35% of MS cases compared to 0% of non-MS cases [48]. Given these promising results we hypothesized that EBV may be involved in the pathogenesis of MS. We aimed at expanding our study to include multiple samples from 122 postmortem MS and non-MS coronal brain tissues to examine for the presence of EBV, determine viral load and viral gene expression, and characterize the phenotype of the infected cells.

## Materials and methods

### Samples

This study was carried out on autopsied human brain tissues received as large coronal slices fixed and preserved in 10% buffered formalin. Tissues were provided by the Rocky Mountain Multiple Sclerosis Centre (RMMSC) tissue bank, University of Colorado (Denver, CO, USA). This study was approved by Al Ain Medical District Human Research Ethics Committee (application number AAMD HREC 12/95).

Of the 122 cases included in the study, 101 had a clinical history of MS, which was confirmed upon postmortem examination. The 21 non-MS control cases, 9 had confirmed neuropathologies, namely meningioma, Alzheimer's disease (AD), progressive supranuclear palsy-like tauopathy (PSPT), and ischemic brain injury. The length of tissue fixation varied from 2 to 23 years and the mean postmortem interval from death was 16 h (range: 4–41 h). Demographics of Non-MS and MS cases are summarized in supplementary S1 and S2 Tables, respectively. Clinical and autopsy data of all 122 cases included in this study is provided in supplementary S3 Table. Paraffin blocks from an EBV-infected infectious mononucleosis (IM) tonsil was used as a positive control. The histology of all the cases was reviewed by a consultant histopathologist (SAS). All of the experiments in this study were performed blindly, and thus samples, regardless of whether from MS or non-MS group, were subjected to same testing conditions.

### Sample processing

For molecular studies, a single small piece of tissue (each ~250 mg) was cut from the white matter region and 3 pieces from different regions of the meninges. Tissues were placed in sterile 15-ml tubes filled with PBS for subsequent DNA extraction. For histology work, 3 pieces of tissue (mean dimensions: 1cm x 1.5cm x 1.5cm) were cut from white matter and white matter-grey matter junction and 3 pieces, whenever available, from meninges. Tissues were dehydrated and processed for embedding in paraffin wax. In total, 615 paraffin blocks were studied. All samples, MS and non-MS were studied blindly.

### EBV PCR on brain tissues

DNA was extracted from the white matter and meninges using our phenol/chloroform- based protocol optimized for long-term formalin-preserved archival tissues [48]. The quantity and purity of the extracted DNA was determined using the Nanodrop-1000 spectrophotometer (Nanodrop Technologies, USA) and the 260/280 OD ratio. PCR was performed on 100ng of genomic DNA as previously described [48]. The amplifiable quality of extracted DNA was checked by amplifying a 104-bp fragment of the house-keeping gene, β-globin [48]. For EBV PCR, a 152-bp BamHI W fragment was amplified [49]. For EBV positive control, DNA extracted from EBV infected cell line (B95-8) was used (100ng of genomic DNA). To check for contamination, several negative controls (no template DNA) were included in each PCR run. PCR was also performed for 3 other common human herpesviruses, namely herpes simplex virus 1 (HSV-1), cytomegalovirus (CMV) and human herpesvirus 6 (HHV-6) to assess the possibility of non-selective infiltration of common viruses into the brain. For this purpose, PCR amplification of a 92-bp fragment of HSV-1 [50], 137-bp of HHV-6 [51] and 139-bp of CMV [51] was carried out on randomly selected 16 EBV PCR positive brain samples. PCR was performed using Applied Biosystems thermal cycler 2700; 40 cycles of amplification were used for all primers. While all thermal conditions were kept constant, the annealing temperatures for HSV-1, CMV and HHV-6 primers were 63°C, 62°C, and 53°C respectively. Amplicons were electrophoresed and visualized in 2% agarose gel stained with ethidium bromide.

### Quantitative real-time PCR (qPCR) on brain tissues

Brain samples found to be positive for EBV using conventional PCR, were further tested to determine viral load using quantitative Taqman PCR targeting EBV BamH1 W fragment [52,53]. To estimate EBV copy number, a standard curve was generated using serial dilutions (100, 10, 1, 0.1, 0.01 ng) of Namalwa cell line DNA (an EBV positive Burkitt’s lymphoma cell line carrying 2 copies of EBV/cell). Reactions were run in duplicates and mean Ct values were plotted against EBV copy number. Viral load in our test samples was extrapolated from the standard curve. Each reaction was set in a final volume of 20μl consisting of 1x TaqMan Universal PCR Master Mix, 1x primer-TaqMan probe combination (Applied Biosystems), 50 ng DNA. All samples were run in duplicates in a 40-cycle reaction using Applied Biosystem 7500 real time PCR machine. The sequences of the PCR primers used in this study are given in Table 1.

**Table 1:** Sequences of the PCR primers used in this study.

Genomic fragment	Primers sequence	Product size (bp)	Reference
**β-globin**	F: 5' GAG GTT CTT TGA GTC CTT TGG 3' R: 5' CAT CAC TAA AGG CAC CGA GCA 3'	104	[48]
**EBV**	F: 5' CAC TTT AGA GCT CTG GAG GA 3' R: 5' TAA AGA TAG CAG CAG CGC AG 3'	152	[49]
**EBV-qPCR**	F: 5' GCA GCC GCC CAG TCT CT 3' R: 5' ACA GAC AGT GCA CAG GAG CCT 3' Probe: 5' (6FAM)AAA AGC TGG CGC CCT TGC CTG(TAMRA) 3'	83	[52]
**HSV-1**	F: 5' CAT CAC CGA CCC GGA GAG GGA C 3' R: 5' GGG CCA GGC GCT TGT TGG TGT A 3'	92	[50]
**CMV**	F: 5' CCG CAA CCT GGT GCC CAT GG 3' R: 5' CGT TTG GGT TGC GCA GCG GG 3'	139	[51]
**HHV-6**	F: 5' TTA AAC AGC CGT TGT CAG GG 3' R: 5' GTA TCC CGA CGG CAG AGG TT 3'	137	[51]

### EBER *in situ* hybridization (EBER-ISH) on brain sections

To localize EBV infected cells in the brain, we used EBER-ISH, an extremely sensitive approach that in our hands can localize a single positive cell in a tissue section [54,55]. EBER-ISH was carried out on all 615 paraffin blocks following the detailed protocol previously published [54]. Briefly, deparaffinized 5μm-thick sections were dehydrated and tissue endogenous peroxidase activity was quenched in 0.5% H_2_O_2_ in methanol. Tissues were pretreated with 100μg/ml proteinase K (Cat # P6556, Sigma) for 10 min at 37°C. Sections were incubated with 10μg/ml mixture of antisense EBER1 and EBER2 probes end-labelled with digoxigenin-dUTP, and subjected to brief microwave irradiation followed by overnight hybridization in 42°C oven. Stringency washes were performed in 0.1x SSC in 55°C water bath. Tissues were then incubated with monoclonal mouse anti-digoxigenin (1:2500, clone D1-22, Sigma, US). Ultra-sensitive ABC peroxidase mouse IgG staining kit (Cat# 32052, Thermo Scientific) and Diaminobenzidine tetrahydrochloride (DAB) (Cat# D5637, Sigma) were used for signal detection. Sections were counterstained with haematoxylin and mounted. For each test sample, a second section (consecutive section where possible) was hybridized with a mixture of sense (non-complimentary) EBER probes as a negative control. With each batch of EBER-ISH run, a section from an EBV-positive IM tonsil was included as a positive control. Positive cells per section were counted manually and grouped into 3 categories: ‘+’ (1–49 positive cells), ‘++’ (50–200 positive cells) and ‘+++’ or ‘heavily infected cases’ (>200 positive cells). To determine if there was any correlation between EBV positivity in the brain and immune infiltration, tissue sections were stained with haematoxylin and eosin (H&E) and assessed for degree of inflammatory infiltrate as previously described [56]. Cases were categorized as: 0 (mild infiltration), + (moderate infiltration) and +++ (heavy infiltration).

### Immunohistochemistry (IHC) to characterize EBV gene expression and phenotype of EBV infected cells

Heavily infected cases, as determined by EBER-ISH, were immunostained for a number of viral and cellular markers to determine EBV gene expression and identify the phenotype of the infected cells in the brain. Deparaffinized sections were rehydrated, incubated in 0.5% H_2_O_2_ in methanol, and subjected to heat-induced antigen retrieval in Tris-HCl, pH 9.0 [57]. Tissues were blocked with 5% bovine serum albumin (BSA) in PBS-0.1% Triton X-100, followed by overnight incubation at 4°C with one of the following primary antibodies: mouse monoclonal anti-BZLF1 (1:100, clone BZ1, Santa Cruz Biotechnology, US), mouse monoclonal anti-EBNA1 (1:25, clone D810H, Thermo, US) [58], mouse monoclonal anti-CD3 (1:100, clone PC3/188A, Santa Cruz Biotechnology, US), mouse monoclonal anti-CD19 (1:100, clone LE, Thermo, US), mouse monoclonal anti-CD20 (1:500, clone L26, Thermo, US), mouse monoclonal anti-GFAP (1:5000, clone VPG805, Vector), or goat polyclonal anti-Iba1 (1:2000, Abcam, Cambridge, UK). After overnight incubation in primary antibody, sections were incubated with appropriate secondary antibodies followed by avidin-biotin peroxidase complex and the color of the reaction was developed using DAB. Sections were counterstained with hematoxylin.

To identify the phenotype of EBV infected cells, we combined IHC and EBER-ISH staining. This was carried out by first performing IHC for a cellular marker using the DAB system, followed by EBER-ISH using anti-digoxigenin conjugated to alkaline phosphatase and NBT-BCIP substrate (Cat# ab7468, Abcam, UK,).

### Data analysis

As mentioned above, presence of EBV was examined using PCR and EBER-ISH on a total of 1055 specimens from 101 MS and 21 non-MS cases. Any case that was found to be positive by either PCR or EBER-ISH in at least one specimen was considered to be EBV positive. Thus, the cases that were negative for EBV by both methods and in all specimens from these cases were considered to be EBV negative. Data was expressed using Barr charts.

## Results

### Detection of EBV genome in MS white matter and meninges

**Fig 1:**
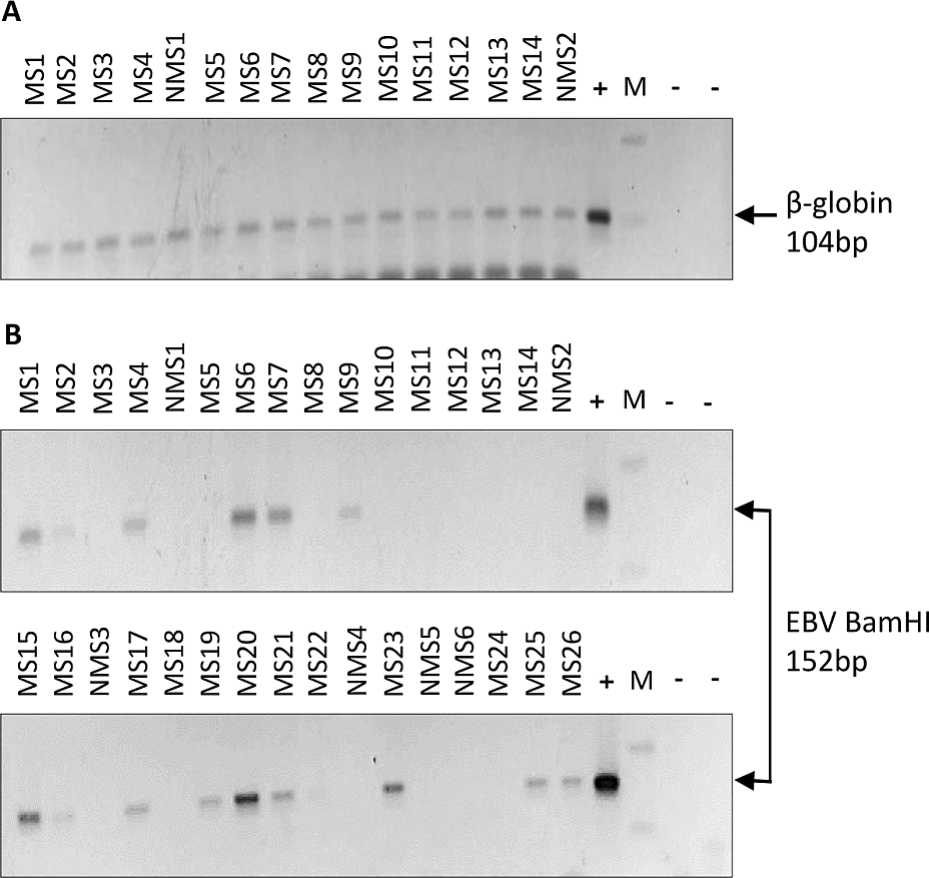
PCR was performed on white matter and meningeal DNA. (A) PCR amplification of Beta-globin (104bp) was used to assess amplifiability of the extracted DNA. (B) Samples that successfully amplified the house-keeping gene were screened for EBV by PCR. In this representative gel, a total of 32 MS and non-MS cases were blindly tested for EBV by PCR. 15/26 MS cases, but 0/6 non-MS controls were found to be EBV positive. [MS: MS case; NMS: non-MS case; M: 100 base DNA marker; (-): negative control (no DNA template);(+): positive control (B95-8 DNA)].

For screening of EBV in the brain, DNA was extracted from both white matter and meninges. All 122 cases had 1 replica from the white matter and 1–3 replicas from the meninges. Only samples that had successful amplification of β-globin (indicative of DNA suitability for PCR) were further tested for EBV (Fig 1). In the MS group, 42 (out of 101) cases were positive for EBV in white matter, and 47 (out of 101) positive in meninges. Meninges showed more positivity for EBV than the white matter, probably due to the fact that more replicas (1–3) from meninges were examined compared to the 1 replica/case from the white matter. Overall, 65/101 (64%) MS cases were EBV positive by standard PCR. It should be noted that 5/21 non-MS cases were also found to be EBV positive. Further examination revealed that these cases had prominent neuropathology, such as dementia, AD, meningioma and PSPT (see supplementary S3 Table for further details). Thus, these non-MS controls were not truly ‘normal brains’.

Analysis of multiple meningeal replicas indicated that 31 cases were positive in one replica, 18 in 2 replicas and a single case was positive in all 3 replicas. This implies that where we search for the virus makes a difference in determining whether a sample is positive or not.

**Fig 2:**
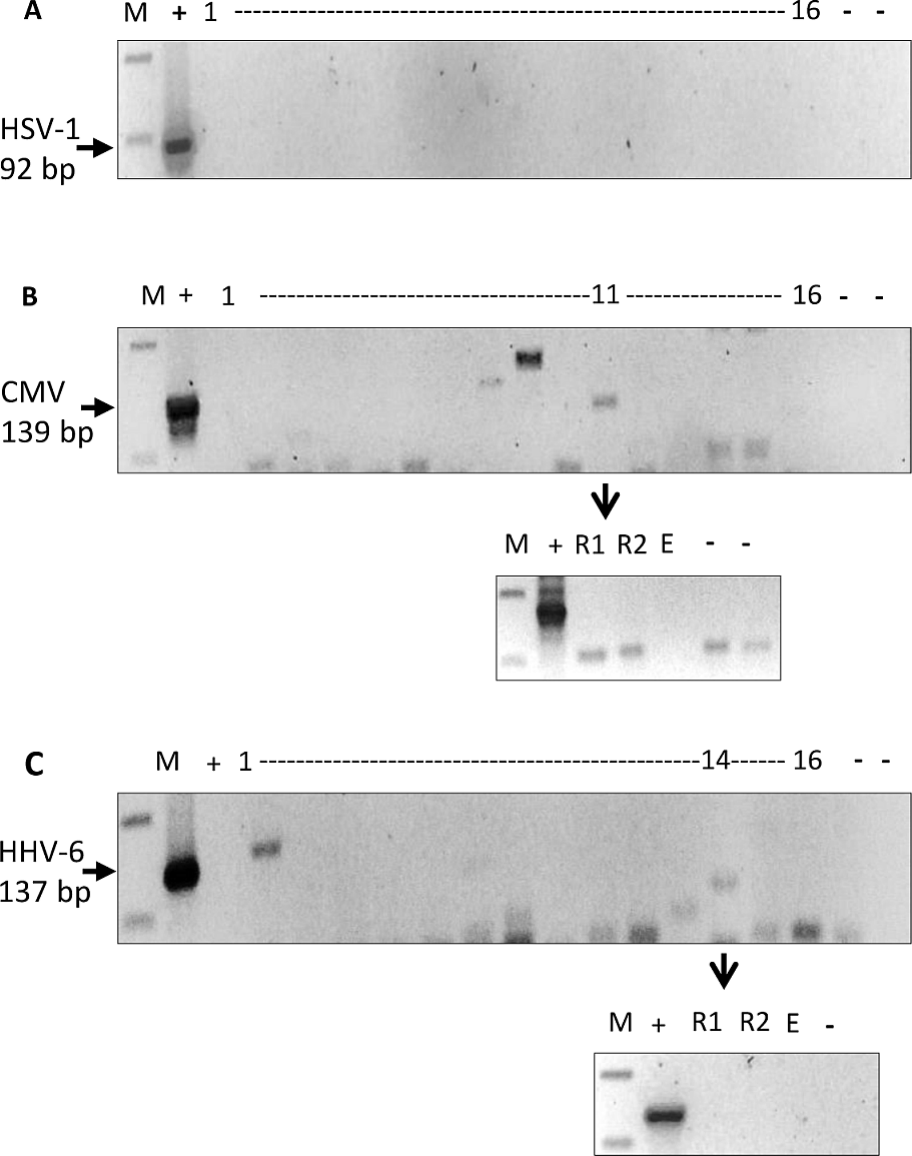
PCR amplification for 3 common herpesviruses. PCR for HSV-1, CMV and HHV-6 was performed on randomly selected 16 white matter DNA that were EBV PCR positive. (A) None of the 16 DNA samples tested were positive for HSV-1 92bp fragment. (B) Similarly, none of these samples amplified CMV 139bp fragment. One sample (case 11) gave an amplification product of the expected size, but on repeat (R1 and R2), no amplification was seen. (C) All 16 DNA samples were also negative for HHV-6 137bp fragment. Sample 14 gave a weak band, but once again on repeating (R1, R2), it was found to be negative. [M: 100bp DNA marker; (-): negative control; (+): positive control DNA; E: empty well].

To determine if EBV presence in the brain was specific and selective to this virus, we randomly selected 16 EBV PCR positive white matter tissues and performed PCR for 3 common herpesviruses, HSV-1, CMV and HHV-6. All samples tested were negative for these viruses (Fig 2), supporting a more specific role for EBV in MS rather than just a mere coincidental infiltration. It is also possible that the absence of these viruses in these cases may be due to the relatively lower sensitivity of the herpesvirus PCR compared to the EBV PCR which targets a reiterated BamH1 W sequence in the viral genome.

### Quantification of EBV viral load in the brain

All of EBV PCR positive samples were further tested to determine viral load. Measurable viral load, ranging from 157 to 16,823 copies (mean: 1,556 ± 2,608) per μg DNA was found in 40 white matter DNA samples and one meningeal DNA sample. The difference in the EBV positivity by qPCR between the white matter and meningeal samples could be explained by the inherently different cellular nature and content of the two tissues. Although both conventional and qPCR consisted of 40 cycles of amplification, for qPCR, amplification at or above mean Ct value of 34 (less than 10 EBV copies/100ng DNA) was considered too low to be deemed positive. Samples were divided into three categories depending on EBV copy number, namely low (100–999), moderate (1,000–1,999) and high (2,000-plus) copies per μg DNA. Majority of samples had low to moderate EBV load, and only 3 samples were considered to have high viral load (Table 2).

**Table 2:** Determination of EBV copy number in white matter DNA. EBV viral load (copies per μg DNA) was grouped into 3 categories: Low: 100 to 999; moderate: 1000 to 1999, and high: ≥2000/μg DNA. Of the 40 cases with measurable viral load, 3 cases were from non-MS control group. These 3 non-MS cases had low viral load, ranging from 180 to 788 viral copies/μg DNA.

EBV viral load	Number of cases	Median EBV load	Mean EBV load (±SD)
**Low**	16	515	525±279
**Moderate**	21	1,248	1,431±320
**High**	3	4,927	7,923 ±7,844
**Total**	40	1,157	1,556 ± 2,608

### Localization of EBER positive cells in MS brain

**Fig 3:**
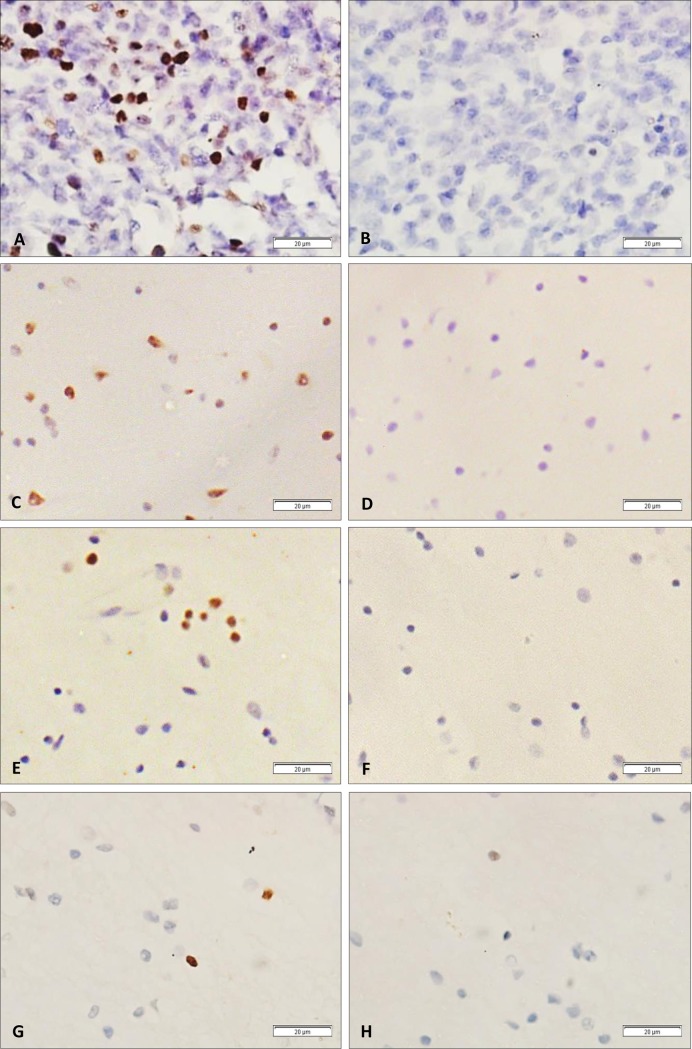
EBER-*in situ* hybridization (EBER-ISH). (A) Tonsil sections from a case of EBV positive infectious mononucleosis (IM). Strong nuclear EBER positive staining (brown) can be seen in cells scattered throughout the tissues. (B) By contrast, section from the same IM case incubated with EBER sense (non-complementary) probes, was clearly negative. (C-H) EBER-ISH on white matter sections from 3 different MS cases representing (C) heavy (+++), (E) moderate (++), and (G) low infection (+). For each case, a section for negative control (using sense probe) was included (D,F,H).

A total of 615 paraffin blocks from 122 cases (101 MS and 21 non-MS), representing different regions were tested for EBV using EBER-ISH to determine EBV infected cells and their location within the brain. Tonsil from an EBV positive case of IM was used as a positive control (Fig 3A). To ensure probe specificity, for each tissue sample, a second section (consecutive section where possible) was hybridized with a mixture of sense (non-complimentary) EBER probes as a negative control (Fig 3B).

EBER *in situ* hybridization showed EBV positive cells in 83/101 (82%) MS cases; 80/101 were positive in the brain parenchyma, and 60/101 in meninges. EBV was also detected in 5/21 non-MS neurological cases. Depending on the number of EBER positive cells within the brain parenchyma (Fig 3), cases were divided into 3 categories; ‘+++’ containing over 200 positive cells (Fig 3C), ‘++’ containing 50 to 200 positive cells (Fig 3E), and ‘+’ containing less than 50 positive cells (Fig 3G). This categorization was made to assess and compare the distribution of EBV positive cells in different cases and between sequential sections of the same tissue block from the same case. While 40/101 cases fell into the lowest category, and 25/101 into the middle category, 18/101 cases were considered heavily infected falling under ‘+++’ category. In the category ‘+++’ group, EBER-ISH positive cells were seen widely distributed within the sections in a scattered fashion (Fig 3C). No EBER-ISH positive clusters were seen in any of the cases. In the category ‘+’ group, only occasional EBER-ISH positive cells were seen (Fig 3G). Moreover, the distribution of EBER positive cells was not uniform in any given case and varied from section to section. This observation emphasizes the importance of screening multiple sections and multiple blocks from each case. Of note, the case with the highest EBV load, according to qPCR data, was also found to be heavily infected using EBER-ISH. The EBER-positive cells in this case were scattered throughout the section, similar to the pattern of distribution seen in other heavily infected (category +++) cases. Furthermore, multiple white matter and meningeal sections were tested from this case and consistently found to be EBV positive by EBER-ISH (see supplementary S1 Fig). Unfortunately, due to the lack of available information on these cases, correlation of EBER positivity and disease subtype, lesion activity, or ethnicity could not be assessed.

Histopathologic examination of the meningeal sections revealed relatively intact fibrous architecture with minimal to moderate infiltration and negligible amount of lymphoid aggregates in most of the examined sections. However, in cases where considerable cellular content was present within the meninges, occasional EBER positive cells were detected. Interestingly, cases which were EBER-ISH positive in meninges, were almost always positive in white matter as well, but not vice versa. The lower level of EBV positivity seen in meninges using EBER-ISH could be attributed to the limited presence of meningeal tertiary lymphoid structures in our samples, which would be the source of EBV-infected B-cells [59]. The presence of B-cell follicles is thought to be linked to progressive MS and cases with prominent subpial grey matter pathology [45,47,60]. Due to lack of clinical data, we cannot rule out the possibility that most of our cases were not progressive disease, hence the lack of meningeal infiltration.

**Fig 4:**
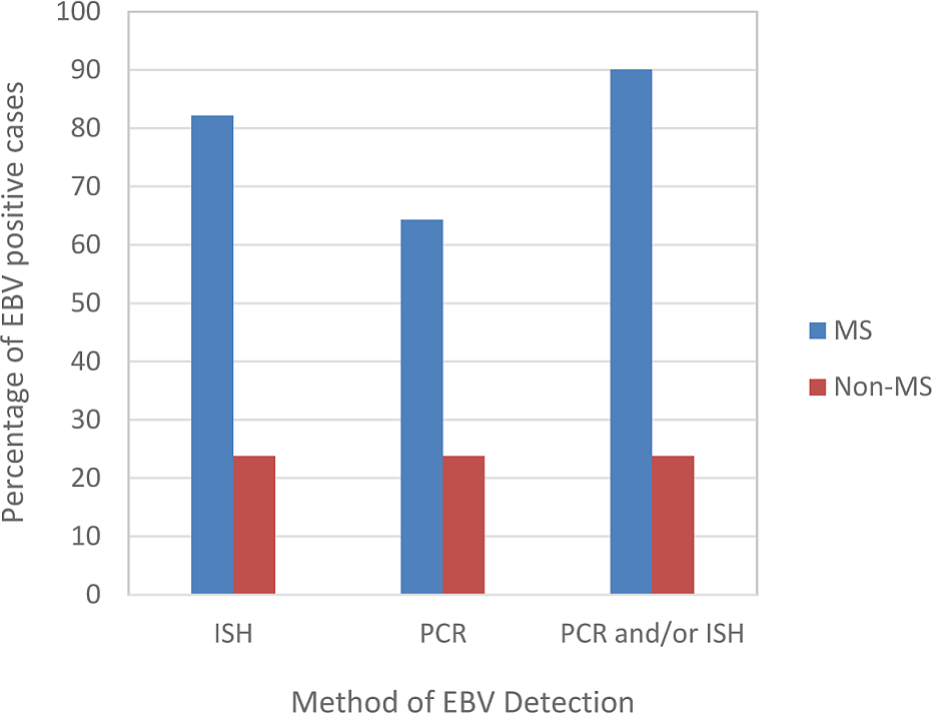
Analyses of EBV positivity in 101 MS and 21 non-MS cases using PCR (440 samples) and EBER-in situ hybridization (615 samples). By EBER-ISH 82% (83/101) of MS cases, but only 24% (5/21) of non-MS cases were EBV positive. By PCR, 64% (65/101) of MS and 24% (5/21) of non-MS cases were positive. Overall, 90% (91/101) of the MS cases were positive for EBV by EBER-ISH and/or PCR. The 5/21 non-MS cases positive by PCR were also positive by EBER-ISH.

Overall, 91/101 (90%) of MS cases were EBV positive by PCR and/or EBER-ISH compared to only 5/21 (24%) non-MS cases (Fig 4). Morover, 58/101 (57%) of the MS cases were positive by both PCR and EBER-ISH.

### Characterization of EBV gene expression in the brain

**Fig 5:**
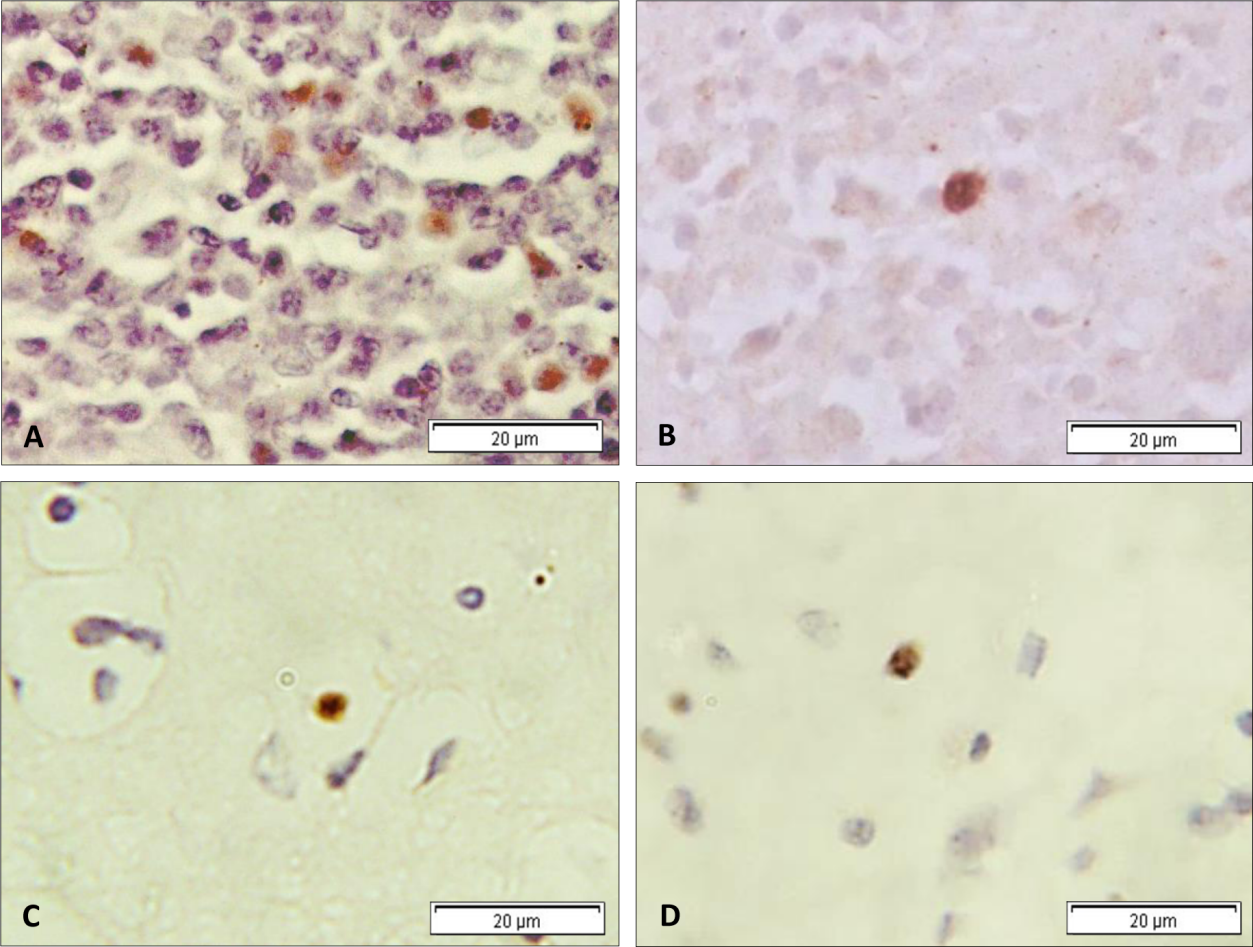
Immunohistochemistry for EBV proteins. Expression of EBNA1 (A) and BZLF1 (B) in EBV-positive IM tonsil. The latent protein EBNA1 was more frequently detected in heavily EBV infected MS cases (C) than the immediate early lytic protein BZLF1 (D). Only occasional BZLF1 positive cells were observed.

Among the 83 EBV infected MS cases, we found 18 to be heavily infected; distinguished by >200 EBER-ISH positive cells. To determine EBV gene expression in these cases, we immunostained for the latent protein EBNA1, and the immediate early lytic protein BZLF1 (Fig 5). Fourteen out of 18 (~78%) of the heavily infected cases were positive for EBNA1 expression in scattered cells ranging in number from 5 to 19 (approximately 1–10% of EBER positive cells) (Fig 5C). In contrast to EBNA1, BZLF1 expressing cells were seen in only 3 (~17%) of these cases. Moreover, the number of positive cells did not exceed more than 1–2% of total EBER-ISH positive cells (Fig 5D). Similar to EBER positive cells, EBNA1 positive and BZLF1 positive cells were scattered and singularly distributed (supplementary S2 Fig).

### Identification of the phenotype of EBV infected cells in the brain

In general, EBER positive cells had lymphocyte-like morphology. Occasionally, positive cells were noted which did not have the typical lymphocyte appearance. H&E staining on heavily infected cases revealed hypercellularity ranging between mild to moderate infiltration. On investigation of which immune cells prevailed in our heavily EBV infected cases, we found a lack of cells expressing CD3, CD19 and CD20. These observations were consistent with the findings reported in the original autopsy histologic examination of these cases. Three cases however, did show the presence of CD3^+^ T-cells and CD20^+^ B-cell in the parenchyma. In one of these cases, considerable level of expression of these cellular markers was noted. This case contained prominent CD20 positive perivascular cuffing with EBER positive cells distributed in and near to the inflamed blood vessels.

**Fig 6:**
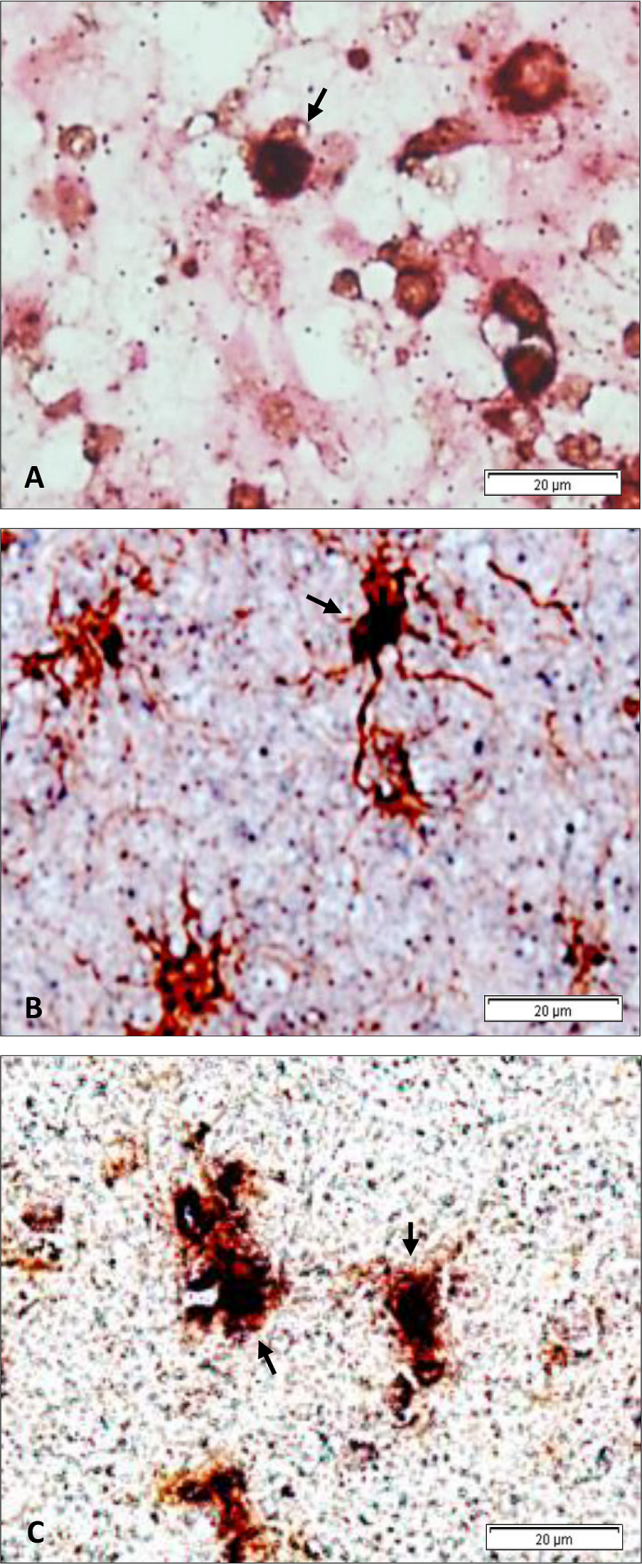
Double staining for EBV and cellular markers. Double staining for EBV (EBER-in situ hybridization: dark blue staining) and different cellular markers (immunohistochemistry: brown) in the white matter of 3 different heavily infected MS cases. The pattern of double-staining seen in these 3 cases is representative of that seen in other double-positive cases. (A) EBV and CD20 (B-cell marker), (B) EBV and GFAP (astrocyte marker), (C) EBV and Iba1 (microglia marker). Double positive cells are indicated by the arrows.

To elucidate the identity of EBER expressing cells, we performed double staining for cellular markers and EBER-ISH. Double staining procedure was first validated using sections from an EBV positive IM tonsil. Double staining on the single MS case which had significant number of CD20 positive perivascular cuffs (supplementary S3 Fig), revealed that some cells were positive for both CD20 and EBV (Fig 6A), indicating that at least some of the EBV infected cells were likely to be B-lymphocytes. To determine if cells other than B-cells may also be harboring the virus, we performed double staining for EBV and Iba1 (marker for microglia), EBV and CD68 (marker of macrophages) and EBV and GFAP (marker of astrocytes). We performed double staining on 18 EBV heavily infected cases. In contrast to CD68, Iba1 and GFAP expressing cells were frequently seen in most of these cases. Remarkably, and to our surprise, 11/18 and 7/18 cases were found to be double positive for EBV/GFAP (Fig 6B) and EBV/Iba1 (Fig 6C), respectively. The number of EBV infected microglia and astrocytes were only 10–15% of the overall EBV (EBER-ISH) infected cells.

## Discussion

An accumulating body of data points to EBV playing a role in the pathogenesis of MS [6–9]. However, the link between EBV and MS is not universally accepted since some studies have failed to consistently find the virus in MS brains [37,43]. It has been argued that these conflicting results are probably due to differences in the tissue samples examined and the sensitivity and specificity of the techniques used [6]. We believe that the extent and degree to which tissues are examined for the presence of EBV is also an important contributing factor to the differences observed. Here we present the most comprehensive analysis of over 1000 brain specimens from 122 MS and non-MS cases for the presence of EBV. We have used multiple highly sensitive and specific approaches for the detection of EBV; PCR for screening EBV in brain tissues, our highly sensitive and specific EBER-*in situ* hybridization (EBER-ISH) for the localization of viral infected cells in the brain tissues, and immunohistochemistry (IHC) to examine the expression of EBV proteins EBNA1 and BZLF1. Additionally, we used double staining approach, EBER-ISH combined with IHC to determine the cellular phenotype of the infected cells. Our findings indicate that EBV is present in brain of most 90% (91/101) of MS cases as determined by EBER-ISH and/or PCR. The fact that we did not find any evidence for the presence of other common human herpesviruses, namely HSV-1, CMV and HHV-6 in any of the cases, indicates that the presence of EBV in MS is likely to be selective and specific. Although we found the presence of EBV in a small fraction of non-MS controls (5/21 cases), these controls were not ‘truly normal’ brains and thus, the significance of the presence of EBV in these cases remains to be determined, as does the possible link between the presence of EBV, neuroinflammation, and neurodegeneration.

Consistent with some previous reports, we also observed evidence of EBER positive cells in an MS case where perivascular regions were infiltrated with CD20 positive B-cells [46,61]. Previous reports have indicated that the presence of B cell-like follicles is a common occurrence in MS lesions and meninges, and could be the reservoir for EBV in the inflamed CNS [38,47,62]. It has been reported that meningeal ectopic follicles occur in about 30–40% of MS cases, particularly the progressive types of MS [63]. These follicles may be associated with subpial demyelination, cortical atrophy and increased activation of microglia leading to grey matter injury [56,64,65]. However, the meninges in most of our cases did not have any prominent lymphoid infiltration. It is possible that some of these discrepancies are due to the heterogeneous dissemination of meningeal immune infiltrates and ectopic B cell follicles [59,60,66]. These tertiary structures are thought to be dependent upon disease length and subtype [47,64]. Differences in the observations, could also be due to variations in sampling and tissue processing.

In this study, EBV infected cells were directly visualized in the brain of 82% of MS cases by EBER-ISH. EBER-ISH is the ‘gold standard’ for the detection of EBV in paraffin sections and the technique is very sensitive and specific [6,67–71]. In our hands, using this technique we have been able to identify a single EBV positive cell in a section [54,55,72]. Although most of our MS cases were EBV positive, the level of EBV infection was low to moderate by both EBER-ISH and qPCR. Despite this level of infection, the direct demonstration of EBV infected cells in the brain of MS cases is significant and could imply a potential role for EBV in the pathogenesis of the disease. Furthermore, 18/101 (18%) of MS cases were heavily infected with EBV (had over 200 EBER positive cells/section). These heavily infected cases were subjected to IHC for determination of EBV gene expression. Our data indicates that both EBNA1 and BZLF1 are expressed in EBV positive MS cases, although only occasional cells express BZLF1. The distribution of EBER positive cells, EBNA1 positive cells and BZLF1 positive cells was scattered rather than aggregated in clusters.

The findings in this comprehensive study support a number of previous reports indicating that EBV is present in a significant proportion of MS cases [45–47]. We previously reported a spatial relationship between EBER expressing cells and IFN-α over-secreting cells; both phenotypic traits were associated with active lesions [46]. In the study by Serafini et al [45], EBV positive cells were found in virtually 100% (21/22) of MS cases, and infected cells were shown to accumulate in ectopic B cell follicles in the cerebral meninges [46]. The same group also reported the presence of EBV infected cells expressing viral lytic markers, closely associated with cytotoxic CD8+ T cells [26]. Although in our study we did not find distinct EBV clusters or follicles, we did however find that the virus was transcriptionally active in the infected cells. In contrast to our study and those mentioned above, some reports have failed to find the presence of EBV in MS brains [37,44,73]. Hence, the association between EBV and MS remains controversial and warrants further investigations.

Although EBV is a highly B-cell tropic virus, our findings suggest that in brain tissues of MS cases, cells other than B-cells can also be infected. Almost all of the heavily infected cases in our study showed only limited expression of CD3, CD19, CD20 or CD68. Coexpression of EBERs and CD20 in one case with significant inflammatory cellular content, suggested that EBV infects CD20 positive B cells. By contrast, cells expressing GFAP and Iba1 were more commonly detectable. The amoebic form taken by virtually all microglia seen in our cases indicated that these cells were in an activated state. EBER signal was detected in a small proportion (~10–15%) of these activated microglia and astrocytes. Consequently, ~85–90% of EBER expressing cells were of indeterminate phenotype. Active microglia were shown recently to stimulate a subtype of astrocytes that can be toxic to neurons and myelin-producing oligodendrocytes [74]. Where EBV fits in this inflammatory cycle has to be investigated. Also, how EBV enters microglia and astrocytes is unknown. EBERs have been shown to be excreted from EBV infected cells via exosomes [75] and trigger TLR3-mediated inflammatory cascade [76]. Thus, occasional uptake of EBERs by neighboring cells, such as microglia and astrocytes, could take place in the absence of bonafide viral infection [77]. Moreover, it has been proposed that EBV may trigger reactivation of MS-associated retroviral elements (HERV-W) which in turn overexpress env protein in microglia and astrocytes [78]. Additionally, astrocytes in MS have been reported to express BAFF, which prolongs the survival of a subset of infiltrating B cells [79]. A recent study in marmoset model of experimental autoimmune encephalomyelitis (EAE) suggested that EBV infection of B cells disrupts the homeostatic cell-to-cell communication that normally occurs between B and T cells, polarizing T cells towards the self-specific inflammatory phenotype [8]. Our study was not designed to examine the possible mechanisms by which EBV infection may contribute to the demyelination, neuroinflammation and neurotoxicity associated with MS. Thus further studies are needed to look at how EBV infection may link to these pathological hallmarks of MS.

In conclusion, this study supports a role for EBV infection in MS, as both EBER-ISH and PCR revealed preferential, but scattered and low level of EBV infection in the brain of most MS cases. Thus, without meticulous and thorough examination, low level of EBV positivity could be easily missed, leading to underestimation of EBV positivity in MS. Our data also suggests that EBV may infect more than one cell type in MS, including microglia and astrocytes. However these findings need to be verified and the possible link between the presence of EBV, neuroinflammation, and neurodegeneration remains to be investigated.

## Supporting information

S1 Table Summary of demographics of non-MS control cases. Age at death in years; expressed as mean± standard deviation, with median value in brackets.
(PDF)

S2 Table Summary of clinical characteristics of MS cases. Age at death, age at disease onset, and duration of disease in years; expressed as means ± standard deviation, with the median value in brackets.
(PDF)

S3 Table Clinical and autopsy data of 101 MS (MS) and 21 control cases (Ctrl). Age is in years, unless otherwise stated in the table. F: Female, M: Male, MI: Myocardial infarction, AD: Alzheimer's disease, GI: gastrointestinal, FHF: Fulminant hepatic failure, HSV: Herpes simplex virus, DIC: Disseminated intravascular coagulation, SPMS: secondary progressive multiple sclerosis, RRMS: relapsing-remitting multiple sclerosis, PE: pulmonary embolism, PPMS: primary progressive multiple sclerosis.
(PDF)

S1 Fig EBER-ISH staining in the case with the highest viral load as determined by qPCR. Numerous EBER-positive cells can be seen scattered in the section using antisense probe (A), but not with sense probe (negative control) (B).
(PDF)

S2 Fig BZLF1 immunohistochemistry in 2 separate cases of MS brains. Occasional, but very clearly positive cells were seen scattered in the section.
(PDF)

S3 Fig Immunohistochemistry staining for CD20. (A) IM tonsil and (B&C) MS brain. In contrast to IM, in MS brain, CD20 positive B-cells were limited in number and often seen in clusters.
(PDF)
