# Supplementary material for: Epstein-Barr virus is present in the brain of most cases of multiple sclerosis and may engage more than just B cells
Source: PLoS One. 2018 Feb 2;13(2):e0192109. doi: 10.1371/journal.pone.0192109 (PMC5796799; doi:10.1371/journal.pone.0192109)
Supplement: S1 Table — Age at death in years; expressed as mean± standard deviation, with median value in brackets. (PDF) [file pone.0192109.s001.pdf]

S1 Table. Summary of demographics of non-MS control cases. Age at death in years; expressed as mean± standard deviation, with median value in brackets.

Control cases	Nº of cases	Mean age at death
**Males**	15	51±27 (55)
**Females**	5	51±11 (52)
**Total**	21	51±24 (55)
