# Supplementary material for: Epstein-Barr virus is present in the brain of most cases of multiple sclerosis and may engage more than just B cells
Source: PLoS One. 2018 Feb 2;13(2):e0192109. doi: 10.1371/journal.pone.0192109 (PMC5796799; doi:10.1371/journal.pone.0192109)
Supplement: S2 Table — Age at death, age at disease onset, and duration of disease in years; expressed as means ± standard deviation, with the median value in brackets. (PDF) [file pone.0192109.s002.pdf]

S2 Table. Summary of clinical characteristics of MS cases. Age at death, age at disease onset, and duration of disease in years; expressed as means ± standard deviation, with the median value in brackets.

MS cases	Nº of cases	Mean age at death	Mean age at MS onset	Mean duration of MS
**Males**	33	60±13 (59)	30±12 (35)	18±9 (17)
**Females**	68	65±13 (66)	32±13 (29)	27±16 (21)
**Total**	101	63±13 (64)	34±12 (32)	24±15 (20)
