# Supplementary material for: Epstein-Barr virus is present in the brain of most cases of multiple sclerosis and may engage more than just B cells
Source: PLoS One. 2018 Feb 2;13(2):e0192109. doi: 10.1371/journal.pone.0192109 (PMC5796799; doi:10.1371/journal.pone.0192109)
Supplement: S3 Table — Age is in years, unless otherwise stated in the table. F: Female, M: Male, MI: Myocardial infarction, AD: Alzheimer's disease, GI: gastrointestinal, FHF: Fulminant hepatic failure, HSV: Herpes simplex virus, DIC: Disseminated intravascular coagulation, SPMS: secondary progressive multiple sclerosis, RRMS: relapsing-remitting multiple sclerosis, PE: pulmonary embolism, PPMS: primary progressive multiple sclerosis. (PDF) [file pone.0192109.s003.pdf]

S3 Table. Clinical and autopsy data of 101 MS (MS) and 21 control cases (Ctrl). Age is in years, unless otherwise stated in the table. F: Female, M: Male, MI: Myocardial infarction, AD: Alzheimer's disease, GI: gastrointestinal, FHF: Fulminant hepatic failure, HSV: Herpes simplex virus, DIC: Disseminated intravascular coagulation, SPMS: secondary progressive multiple sclerosis, RRMS: relapsing-remitting multiple sclerosis, PE: pulmonary embolism, PPMS: primary progressive multiple sclerosis.

**Case**	**Age**	**Sex**	**Cause of death (COD)/ remarks**
**Ctrl1**	55	F	Extensive end-stage long-standing interstitial lung disease
**Ctrl2**	25 wk	M	Immature lung development
**Ctrl3**	50	M	Multifactorial: severe interstitial lung disease with honeycombing, cor pulmonale, MI
**Ctrl4**	37.5 wk	M	Stillborn infant: Intrauterine foetal death due to placental infarction. Neuro-histopathology revealed upon postmortem investigation
**Ctrl5**	73	M	Acute bronchopneumonia
**Ctrl6**	55	M	Alcohol induced cirrhosis
**Ctrl7**	N/A	M	Dementia with AD
**Ctrl8**	N/A	M	Acute MI
**Ctrl9**	N/A	M	MI with emphysema
**Ctrl10**	N/A	F	Nodular sclerosing Hodgkin's
**Ctrl11**	N/A	M	Progressive dementia with AD
**Ctrl12**	69	M	Respiratory distress due to acute bilateral pneumonia
**Ctrl13**	38	F	Massive GI haemorrhage secondary to idiopathic FHF. Neuro-histopathology revealed upon postmortem investigation
**Ctrl14**	42	M	FHF with HSV hepatitis & haemorrhagic necrosis + DIC
**Ctrl15**	68	M	Definite AD - slowly progressive dementia
**Ctrl16**	49	F	Cryptogenic cirrhosis & hepatitis. Hepatic encephalopathy
**Ctrl17**	50	M	Multi-organ failure secondary to ischaemic cardiomyopathy. Neuro-histopathology revealed upon postmortem investigation
**Ctrl18**	76	M	Ventricular fibrillation secondary to E-Coli septicaemia. Neuro-histopathology revealed upon postmortem investigation
**Ctrl19**	63	F	Widely metastatic ovarian adenocarcinoma stage IIIc. Neuro-histopathology revealed upon postmortem investigation
**Ctrl20**	74	M	Progressive supranuclear palsey-like Tauopathy
**Ctrl21**	N/A	N/A	Meningioma
**MS1**	43	M	Severe chronic active & inactive MS
**MS2**	74	M	Moderately severe chronic inactive MS
**MS3**	79	F	Chronic inactive MS. Arteriosclerosis
**MS4**	52	F	Chronic inactive MS
**MS5**	48	M	Moderately severe chronic active MS
**MS6**	69	F	Chronic inactive MS
**MS7**	74	F	Chronic inactive MS
**MS8**	48	M	Subacute MS: involving cervical spinal cord
**MS9**	57	M	Chronic inactive MS
**MS10**	50	M	Chronic active SPMS
**MS11**	68	F	Moderately severe chronic inactive MS
**MS12**	61	F	Mild chronic inactive
**MS13**	67	F	Moderately severe chronic inactive MS. >20-year course
**MS14**	80	M	Severe chronic inactive MS: affecting spinal cord
**MS15**	58	M	Chronic active smoldering & chronic inactive

**MS16**	77	F	Chronic inactive SPMS: affecting spinal cord
**MS17**	61	F	Severe chronic inactive MS
**MS18**	63	F	Chronic inactive SPMS: severely affecting cervical spinal cord
**MS19**	68	F	Chronic inactive MS
**MS20**	N/A	F	Multiple active & reactivated MS plaques
**MS21**	81	F	Moderately severe chronic inactive RRMS
**MS22**	32	F	Severe chronic progressive MS
**MS23**	46	F	Progressive MS
**MS24**	77	M	Moderately severe chronic inactive MS
**MS25**	77	F	Chronic inactive MS coexisted with AD
**MS26**	78	F	Moderately severe chronic inactive MS (38-year course)
**MS27**	N/A	M	Moderately severe chronic active MS (30-year course) COD: PE
**MS28**	41	F	Chronic inactive MS
**MS29**	50	M	Chronic inactive MS
**MS30**	70	F	Chronic inactive MS
**MS31**	54	M	Chronic inactive MS
**MS32**	87	F	Chronic inactive spinal MS. Arteriolosclerosis
**MS33**	70	M	MS (19-year course)
**MS34**	N/A	M	Moderately severe chronic inactive MS. Arteriolosclerosis, cranial bleed
**MS35**	52	M	Moderately severe chronic inactive MS
**MS36**	67	F	Progressive MS
**MS37**	54	F	Severe chronic inactive MS (25-year course)
**MS38**	69	F	Chronic inactive MS
**MS39**	75	F	Chronic inactive MS: affecting spinal cord. COD: respiratory failure
**MS40**	58	F	Chronic inactive MS (20-year course). Atherosclerosis, arteriosclerosis
**MS41**	60	F	Chronic inactive MS
**MS42**	53	F	Moderately severe chronic inactive MS
**MS43**	50	F	Moderately severe chronic inactive MS
**MS44**	65	F	Very minimal demyelinative MS plaque burden
**MS45**	92	F	Moderate chronic inactive MS. Arteriolosclerosis
**MS46**	59	M	Moderately severe chronic inactive MS. Atherosclerosis
**MS47**	59	M	MS- no further information available
**MS48**	43	M	Chronic inactive with chronic active smoldering MS
**MS49**	59	M	Chronic inactive MS
**MS50**	53	F	MS- no further information available
**MS51**	56	F	Severe chronic inactive PPMS, (40-year course). Idiopathic Parkinsonism
**MS52**	29	F	MS- no further information available
**MS53**	76	F	MS- no further information available
**MS54**	42	M	MS- no further information available
**MS55**	67	M	MS- no further information available
**MS56**	65	F	MS- no further information available
**MS57**	42	M	MS- no further information available
**MS58**	98	M	MS- no further information available
**MS59**	77	F	MS- no further information available
**MS60**	79	M	MS- no further information available
**MS61**	66	M	MS- no further information available
**MS62**	70	F	MS- no further information available
**MS63**	32	F	Chronic inactive noninflammatory RRMS
**MS64**	N/A	F	MS- no further information available
**MS65**	60	F	MS- no further information available
**MS66**	57	F	Chronic active MS
**MS67**	70	M	MS- no further information available
**MS68**	56	F	MS- no further information available

**MS69**	75	F	MS- no further information available
**MS70**	71	F	MS- no further information available
**MS71**	45	M	Severe spinal mixed chronic inactive & active MS (10-year)
**MS72**	57	F	MS- no further information available
**MS73**	66	F	MS- no further information available
**MS74**	75	M	MS- no further information available
**MS75**	59	M	Moderately severe chronic active MS
**MS76**	60	F	MS- no further information available
**MS77**	61	F	MS- no further information available
**MS78**	65	F	MS- no further information available
**MS79**	61	F	Severe chronic active smoldering MS
**MS80**	84	F	MS- no further information available
**MS81**	71	F	MS- no further information available
**MS82**	71	F	MS- no further information available
**MS83**	61	M	MS- no further information available
**MS84**	62	M	MS- no further information available
**MS85**	63	F	Chronic inactive SPMS
**MS86**	74	F	Chronic inactive MS
**MS87**	65	F	MS- no further information available
**MS88**	89	F	MS- no further information available
**MS89**	70	M	MS- no further information available
**MS90**	46	F	MS- no further information available
**MS91**	59	F	MS- no further information available
**MS92**	69	F	MS- no further information available
**MS93**	70	F	MS- no further information available
**MS94**	N/A	F	MS- no further information available
**MS95**	64	F	MS- no further information available
**MS96**	60	F	MS- no further information available
**MS97**	56	M	MS- no further information available
**MS98**	67	F	MS- no further information available
**MS99**	67	F	MS- no further information available
**MS100**	74	F	Chronic inactive SPMS with superimposed relapses
**MS101**	77	F	MS- no further information available
