# Supplementary material for: Epstein-Barr virus is present in the brain of most cases of multiple sclerosis and may engage more than just B cells
Source: PLoS One. 2018 Feb 2;13(2):e0192109. doi: 10.1371/journal.pone.0192109 (PMC5796799; doi:10.1371/journal.pone.0192109)

S1 Fig. EBER-ISH staining in the case with the highest viral load as determined by qPCR. Numerous EBER-positive cells can be seen scattered in the section using antisense probe (A), but not with sense probe (negative control) (B).

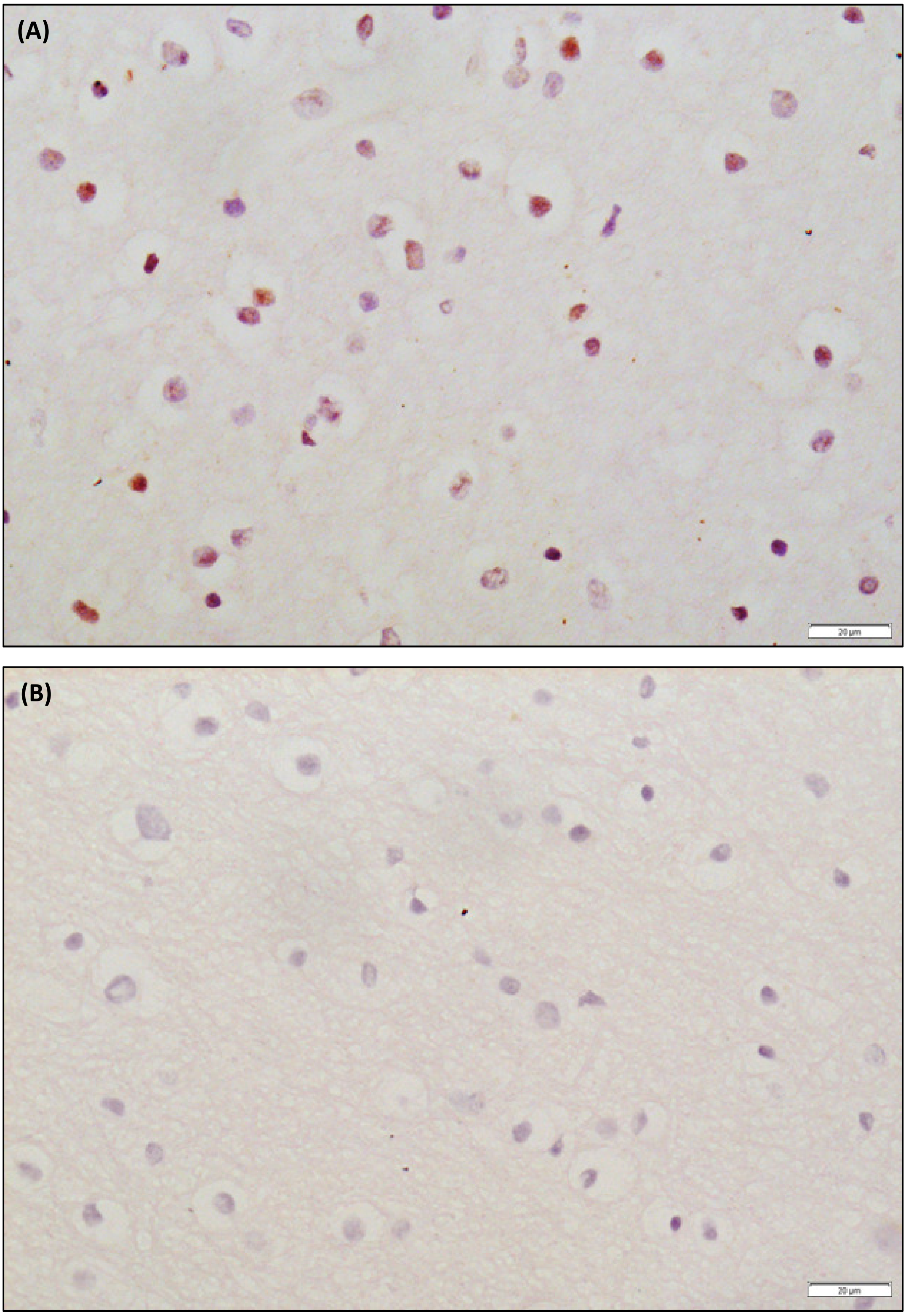

Supplement: S1 Fig — Numerous EBER-positive cells can be seen scattered in the section using antisense probe (A), but not with sense probe (negative control) (B). (PDF) [file pone.0192109.s004.pdf]
