# Supplementary material for: Epstein-Barr virus is present in the brain of most cases of multiple sclerosis and may engage more than just B cells
Source: PLoS One. 2018 Feb 2;13(2):e0192109. doi: 10.1371/journal.pone.0192109 (PMC5796799; doi:10.1371/journal.pone.0192109)

S2 Fig. BZLF1 immunohistochemistry in 2 separate cases of MS brains.
Occasional, but very clearly positive cells were seen scattered in the section.

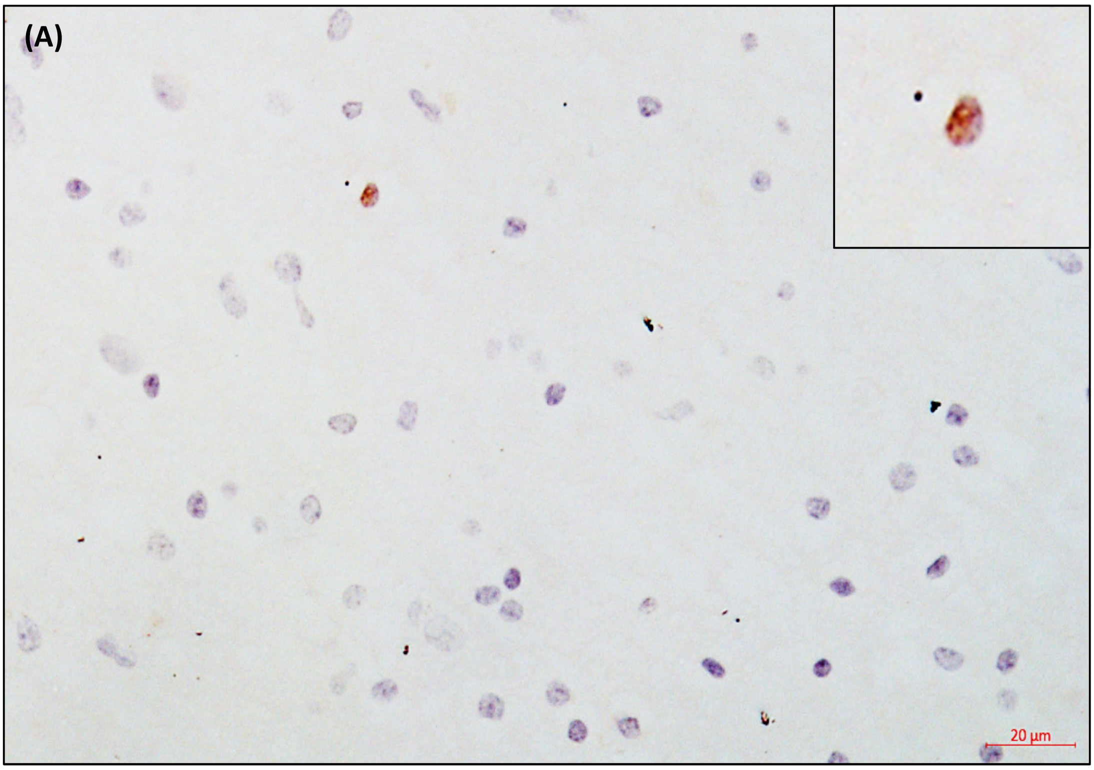

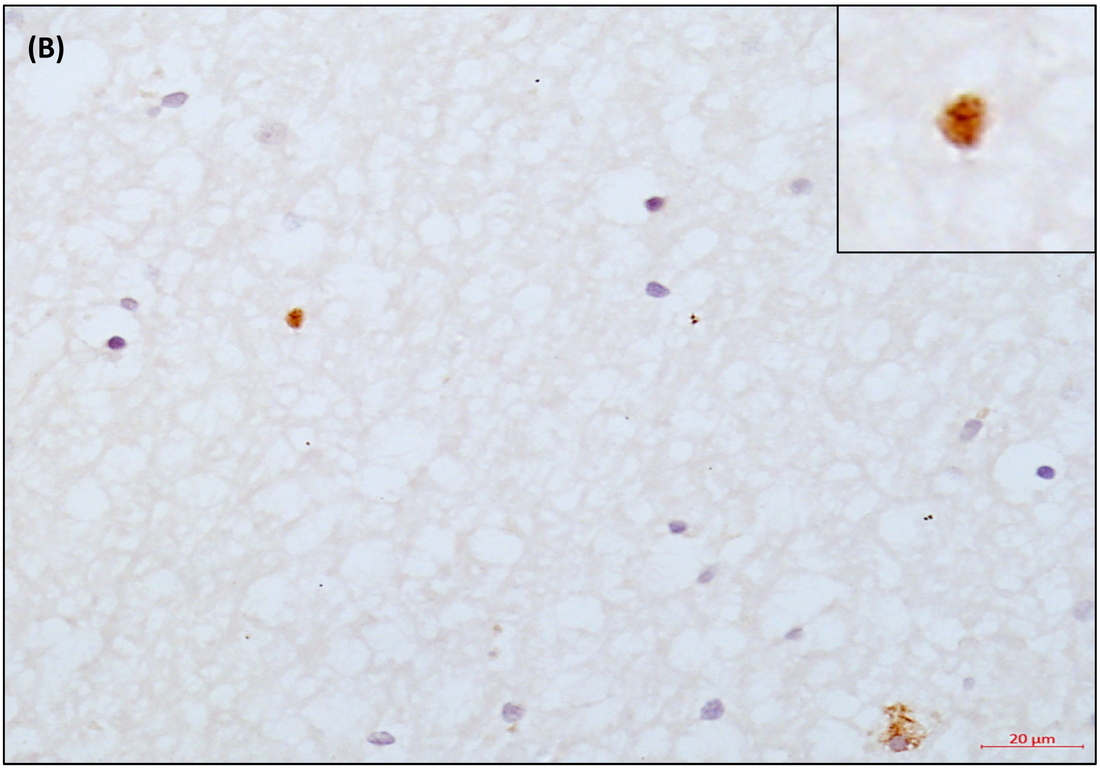

Supplement: S2 Fig — Occasional, but very clearly positive cells were seen scattered in the section. (PDF) [file pone.0192109.s005.pdf]
