# Supplementary material for: Epstein-Barr virus is present in the brain of most cases of multiple sclerosis and may engage more than just B cells
Source: PLoS One. 2018 Feb 2;13(2):e0192109. doi: 10.1371/journal.pone.0192109 (PMC5796799; doi:10.1371/journal.pone.0192109)

S3 Fig. Immunohistochemistry staining for CD20. (A) IM tonsil and (B&C) MS brain. In contrast to IM, in MS brain, CD20 positive B-cells were limited in number and often seen in clusters.

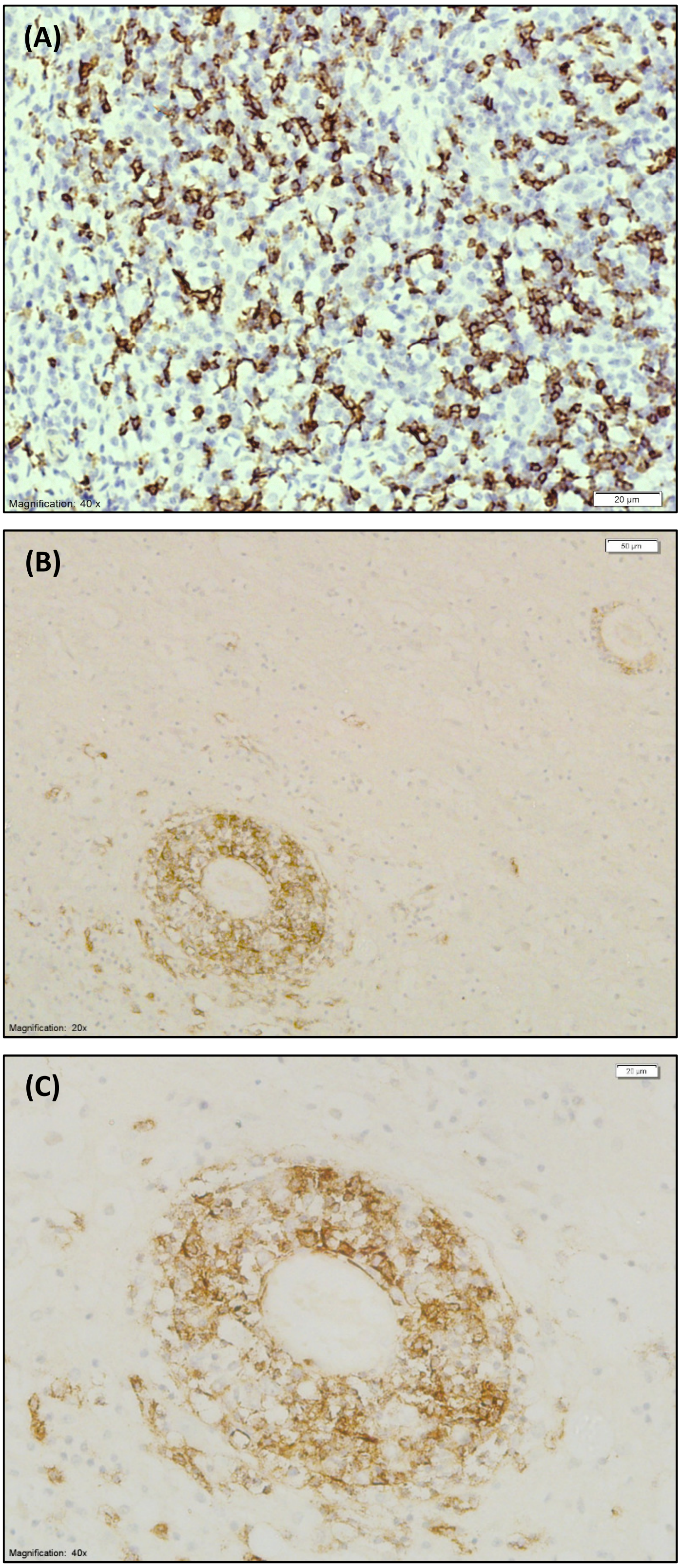

Supplement: S3 Fig — (A) IM tonsil and (B&C) MS brain. In contrast to IM, in MS brain, CD20 positive B-cells were limited in number and often seen in clusters. (PDF) [file pone.0192109.s006.pdf]
